# Magnetic Solid Nanoparticles and Their Counterparts: Recent Advances towards Cancer Theranostics

**DOI:** 10.3390/pharmaceutics14030506

**Published:** 2022-02-25

**Authors:** Mónica Cerqueira, Efres Belmonte-Reche, Juan Gallo, Fátima Baltazar, Manuel Bañobre-López

**Affiliations:** 1 Life and Health Sciences Research Institute (ICVS), Campus of Gualtar, University of Minho, 4710-057 Braga, Portugal; monica.cerqueira@inl.int; 2 ICVS/3B’s—PT Government Associate Laboratory, 4805-017 Guimarães, Portugal; 3 Advanced (Magnetic) Theranostic Nanostructures Lab, Nanomedicine Unit, International Iberian Nanotechnology Laboratory, Avenida Mestre José Veiga, 4715-330 Braga, Portugal; efres.belmonte@inl.int (E.B.-R.); juan.gallo@inl.int (J.G.)

**Keywords:** solid lipid nanoparticles, magnetic nanoparticles, magnetic solid lipid nanoparticles, cancer theranostics, MRI-contrast agents

## Abstract

Cancer is currently a leading cause of death worldwide. The World Health Organization estimates an increase of 60% in the global cancer incidence in the next two decades. The inefficiency of the currently available therapies has prompted an urgent effort to develop new strategies that enable early diagnosis and improve response to treatment. Nanomedicine formulations can improve the pharmacokinetics and pharmacodynamics of conventional therapies and result in optimized cancer treatments. In particular, theranostic formulations aim at addressing the high heterogeneity of tumors and metastases by integrating imaging properties that enable a non-invasive and quantitative assessment of tumor targeting efficiency, drug delivery, and eventually the monitoring of the response to treatment. However, in order to exploit their full potential, the promising results observed in preclinical stages need to achieve clinical translation. Despite the significant number of available functionalization strategies, targeting efficiency is currently one of the major limitations of advanced nanomedicines in the oncology area, highlighting the need for more efficient nanoformulation designs that provide them with selectivity for precise cancer types and tumoral tissue. Under this current need, this review provides an overview of the strategies currently applied in the cancer theranostics field using magnetic nanoparticles (MNPs) and solid lipid nanoparticles (SLNs), where both nanocarriers have recently entered the clinical trials stage. The integration of these formulations into magnetic solid lipid nanoparticles—with different composition and phenotypic activity—constitutes a new generation of theranostic nanomedicines with great potential for the selective, controlled, and safe delivery of chemotherapy.

## 1. Introduction

Cancer is a malignant disease involving uncontrolled and rapid growth of aberrant and nonfunctional cells as a result of epigenetic and genetic modifications. These have the capacity to metastasize to distant organs of the body [1]. This heterogeneous disease ranks as a principal public health concern worldwide [2]. In total, 18.1 million new cancer cases were diagnosed in 2018, whilst 9.6 million deaths were related to the disease. Moreover, a 60% incidence increase in new global cancer cases is expected to occur over the next two decades, according to the World Health Organization (WHO) [3]. 

The main tool for an efficient cancer treatment is an early diagnosis, as according to WHO reports, 30% of patients could have successfully been considered cured if diagnosed at an early stage of the disease. When the tumor is identified early (in the first stages), combinations of surgery, chemotherapy, and radiotherapy are usually viable options as treatments with higher success rates and less side effects [4]. However, the latter occurrence of the symptoms leads quite often to a cancer diagnosis at more advanced stages—stage three or four. Then, the subscripted cancer treatment will be dependent on the type and stage of the tumor/s, in addition to the patient’s condition—older and weaker patients are normally spared treatments due to their aggressiveness—where late diagnosis (and/or surgical tumor inaccessibility) limits the treatment of cancers to chemotherapy and immunotherapy [4].

Several research fields are focused on finding anticancer drugs that achieve a selective phenotypic cytotoxic effect on cancer cells. These should, at the same time, stop or slow down tumor growth whilst being less toxic (or ideally innocuous) to healthy tissues [5]. Chemotherapeutic agents obtain different mechanisms of action depending on their pharmacophore structure and other moieties (its chemical structure). Hence, chemotherapeutics can be classified as alkylating agents (e.g., cisplatin and cyclophosphamide), anti-metabolites (e.g., methotrexate and fluorouracil), anthracyclines with DNA-binding antibiotics (e.g., doxorubicin (DOX)), topoisomerase inhibitors (e.g., etoposide), and microtubule stabilizers (e.g., paclitaxel, docetaxel) [4,6]. Although usually effective, the main drawback of these drugs is their selectivity issues, as they can usually have a phenotypic effect on the much more abundant healthy tissue as well. This can cause short and then long-term health sequels in patients and even death [6,7,8,9]. 

When administered intravenously, chemotherapeutics are systemically distributed and therefore can potentially reach all organs. Given its nature as a blood detoxifier—converting xenobiotics into waste products—the liver is usually specially affected by the non-selective action of the drugs [10]. Systemic distribution also reduces the in situ concentration of the compounds in the tumor area. They may therefore require a higher posology to achieve the desired effect, compromising their narrow therapeutic margins [5,11,12]. The poor pharmacokinetics, specificity, and the generation of cancer multidrug-resistance (MDR) can further reduce their therapeutic margins [5,6,7,13]. Altogether, the treatments available and the current poor success rates associated with them require smart targeted strategies to achieve chemotherapeutic selectivity in addition to better early diagnosis and in situ therapies. 

**Figure 1:**
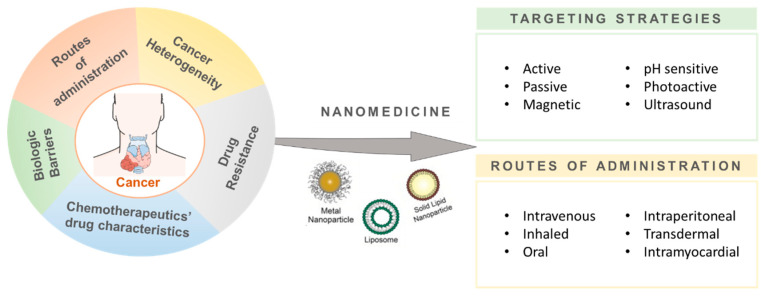
Nanomedicine applications in cancer therapy. Nanoparticles, as drug delivery systems, can enhance the drug targeting to specific body/organ/tissue or even single cancer cells through different targeting strategies (e.g., active/passive, endogenously/exogenously responsive) and different routes of administration (intravenous, oral, or intraperitoneal, among others).

Nanotechnology has evolved into a multidisciplinary field, having revolutionized many scientific and nonscientific areas since 1970, including: applied physics, materials chemistry, chemistry mechanics, robotics, medicine, and biological and electrical engineering [14]. In the bioscience and medicine fields, nanomaterials have a wide range of applications. In cancer therapy, for example, they have been used as diagnostic tools and as drug delivery formulations [15,16]. Their nanoscale size (1–100 nm) makes them ideal candidates for surface nano-engineering and the production of functionalized nanostructures [17]. Hence, they are currently being applied as drug delivery systems (DDS), sensors, and tissue engineering catalyzers, amongst others [18]. Due to their unique physical and optical properties and chemical stability, nanoparticles can grant selectivity to drugs for specific body/organ/tissue targeting and even for individual recognition and targeting of single cancer cells [15,19]. Hence, the nanoparticles’ characteristics can benefit the bioactivity of therapeutic compounds through the reduction of the concentration needed for a certain phenotypic outcome, potentially increasing their therapeutic margins and pharmacokinetic properties and altogether reducing their potential harmful secondary effects in healthy tissues (Figure 1) [14,18,19]. 

Many nanoformulations have been investigated pre-clinically, yet only a minority have advanced to clinic stages [20]. Currently, those approved by the U.S. FDA and European Medicines Agency (EMA) [21] include: Abraxane [22], Doxil [23], and Patisiran/ONPATTRO [24]. These formulations respond to the need for creating new systems that efficiently improve drug selectivity and delivery and that help promote an accurate and safer treatment of cancer. 

Within the cancer field, magnetic nanoparticles (MNPs) have gained interest as highly functionalized tools that can be applied to diagnosis, monitorization, and therapy. Their relative straightforward synthesis, functionalization, purification, and characterization, together with their usually good biodegradability and diagnostic platform potential, confer major advantages for their use in cancer theranostics [25,26,27,28,29,30,31,32,33,34,35]. Recently, NanoTherm^®^, a new platform for the intermittent glioblastoma treatment multiform, was approved by the EMA and evidences the potential these systems have in cancer diagnosis and therapy [36]. Another type of nanoparticle, which is based on solid lipid nanoparticles (SLNs), has also been studied abundantly and is currently applied in cancer therapy. Here, SLNs have been used as a drug delivery system that has the potential to control the release of the loaded chemotherapy and decrease their toxicity with an enhancement of biocompatibility in comparison to inorganic or polymeric nanoparticles [37,38,39,40].

In this review, we provide an overview of recent developments to fight cancer using MNPs and SLNs, alone or in combination, to yield magnetic solid lipid nanoparticles (mSLNs), where we highlight their performance and potential application in diagnosis, drug delivery, and other therapeutic approaches such as magnetic hyperthermia and theranostics. Special focus will be paid to those reports offering results at the advanced preclinical stage, both in vitro and in vivo. 

## 2. Magnetic Nanoparticles

MNPs are being widely studied nowadays in many areas (such as in the biomedical field), because they offer a plethora of opportunities [25]. Their physicochemical properties, superparamagnetic behavior, small size, and capability to promote biological interactions at the cellular and molecular level [25,26] allow MNPs to be employed as drug delivery systems [28,29], magnetic resonance imaging contrast enhancers [30], and hyperthermia inducers [31] for the treatment of cancer. 

A key component of these MNPs is the metal used in their formulations. Thus, they are usually ferrites (MFe_2_O_4_, Ni_a_Zn_(1−a)_Fe_2_O_4_, Mn_a_Zn_(1−a)_Fe_2_O_4_) [41], metal alloys (FeCo, alnico, and permalloy), or iron-based magnetic oxides (hematite (α-Fe_2_O_3_), magnetite (Fe_3_O_4_), and maghemite (γ-Fe_2_O_3_)) [31]. The most commonly used nanoparticles in the biomedical field are superparamagnetic iron oxide nanoparticles (SPIONs), such as Fe_3_O_4_ and γ-Fe_2_O_3_, which present high biocompatibility and lower toxicity compared to other metal structures (e.g., quantum dots, gold nanoparticles, and carbon nanotubes (CNTs) may present lower biodegradation and body-elimination issues [25], together with increased cytotoxicity [32,41]). Their superparamagnetic properties enable a degree of control through the application of an alternating magnetic field (AMF). Here, selective application of the AMF can force the MNPs to generate local heat and promote the direct tumor ablation and/or the drug release into the desired region, ultimately avoiding invasive diagnostic and therapeutic techniques [32,33].

MNP performance is dependent on their composition, morphology, surface coating, and size of the inorganic core, all of which influence their in vivo behavior [25] and potential toxicity [41]. Studies performed in a mouse model with MNPs coated with DMSA (dimercaptosuccinic acid) revealed accumulation in the liver, spleen, and lungs without side effects [34]. Hence, the functionalization of the formulations’ surface with targeted ligands can be a strategy to reduce toxicity in untargeted organs, whilst also increasing the therapeutic efficacy in targeted ones [41].

### 2.1. Magnetic Nanoparticles as Drug Delivery Systems

MNPs have become an interesting vehicle for drug delivery in the cancer therapy field. The MNPs’ design and formulation are part of an interdisciplinary scientific communication where bio-physicochemical interactions between MNPs and cells are optimized. As described by Hung et al. [41], an efficient DDS should: (i) have the capacity to load the appropriate drug/active compound, (ii) improve the biocompatibility, stability, and protect the drug and its bioactivity, and (iii) promote drug delivery at the required site with low toxicity for the healthy cells/tissues, [41]. 

**Figure 2:**
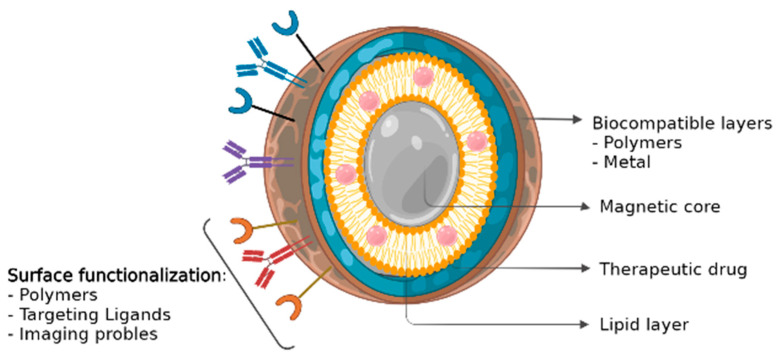
Magnetic nanoparticle (MNP) structure. MNPs are usually constituted by a magnetic core–shell encapsulated by a biocompatible coating [42], where chemotherapeutics are loaded into.

As several MNP production methods have been currently described in the literature, the process can be selected based on the ultimate purpose/objective of the MNPs, which for most is the maximization of the desired phenotypic effect on cancer. On the one hand, the co-precipitation of salts with stabilizing polymer/s, hydro/solvothermal procedures, thermal decomposition, and reverse microemulsions can be considered the traditional methods of MNPs synthesis [27]. On the other hand, newer strategies include microfluidic and biogenic synthesis [36]. In either case, the resulting MNPs are usually constituted by a magnetic core–shell encapsulated by a polymer coating [42], where chemotherapeutics are loaded into (Figure 2). In this manner, the chemotherapeutics also help improve their colloidal stability and pharmacokinetic properties for the posterior systemic administration [43]. The drug loading can also be performed by several methodologies [27,42], although the methodology most employed makes use of the direct encapsulation of the drug or its absorption in the MNPs through physical or chemical interactions. The drug loading efficiency is here dependent on both the properties and compatibilities of the chemotherapy with the MNP and its coating [1]. Hence, MNP coating selection and optimization is the common strategy to effectively load hydrophobic [44] or hydrophilic drugs into the nanoformulations [43]. Different coatings may also have different feasibilities for the formulation administration [45]. Altogether, an effective coating selection will promote the correct loading of the drugs, prevent the nanoparticle agglomeration, and promote an efficient and controlled release at the target site. Typical coatings include lipids, surfactants, or polymers (such as dextran or polyethylene glycol (PEG)). These organic surfactants and polymers enhance the biocompatibility of the nanoparticles and promote opsonization resistance. This expands their systemic circulation time and increases the fraction of nanoparticles that ultimately reach the target (tumor cells) [25,46]. Furthermore, coatings can also lower unwanted cytotoxicity in healthy tissues. For example, for iron oxide nanoparticles coated with PEG, Ruiz and co-workers demonstrated an enhanced residence time and reduced liver and spleen particle accumulation when compared to its uncoated counterpart [35]. 

MNPs loaded with active agents (chemotherapy, DNA, RNA, or antibodies) can further improve their therapeutic effects and margin whilst grating a degree of control over their release in the biological environment [46,47,48,49,50]. Additional selectivity and modulation of the MNP response can be achieved by functionalizing the MNPs [47]. For example, functionalized MNPs have already been prepared to be sensitive to internal metabolic factors of the tumor, such as pH, hypoxia, specific enzymes, and the Warburg effect [25,46,47,48,49]. MNP formulations have also been prepared to be sensitive to an external stimulus to be subjected over the tumor area, such as light or temperature [16,25,30,51]. For the latter, MNPs under either near-infrared (NIR) light or an alternating magnetic field (as the external stimuli) have been found to further modulate the release of the loaded drug [31,52]. Hence, the stimuli can provide an additional level of control over the drug release equilibrium [25,47].

The sum of all of these characteristics makes MNPs very interesting tools for the safe and selective targeting of cancer, in addition to their theranostic capabilities [48,49,50]. 

### 2.2. Magnetic Nanoparticles in Cancer Diagnostics

The WHO’s 2018 world cancer report predicted an increase by 2040 of 60% in cancer incidence. Currently, early detection is the most effective way to increase the probability for successfully overcoming most cancers. These malignancies ideally require a non-invasive, fast, and precise diagnostic system able to provide the position, size, and characteristics of the main tumor, in addition to that of other metastatic bodies [53]. 

A diagnostic tool used in clinic for tumor detection is magnetic resonance imaging (MRI). MRI is a non-invasive, safe, and painless technique that uses magnetism and radio pulses to produce images of different internal tissues and organs from different angles and perspectives. The result is usually a clear depiction of soft tissues, including some tumors [41].

MRI is based on the properties of some atoms to absorb energy in the form of radio waves when under a magnetic field. Such an event causes a spin polarization that can induce a signal in a radio frequency coil that can then be detected by a nearby antennae/detector. Usually, hydrogen nuclei consisting of a single proton are used to create the signals. Hydrogen is naturally abundant in all forms of life and hence can be used to create a macroscopic polarization of hydrogen-rich tissues (rich in water and fats). The pulses of radio waves excite the nuclear spin energy transition whilst the magnetic field gradient localizes their polarization in space. After the excitation, the technique measures the relaxation of the hydrogen in the longitudinal (*T*_1_-spin-lattice relaxation) and transverse planes (*T*_2_-spin-spin relaxation) [33,53,54]. The image formed here is dependent on the tissue’s local atomic density and the association of hydrogen to other atoms. Furthermore, the pulse sequence can generate different contrasts between tissues, as can specific agents that increase the capabilities of MRI. These agents shorten the relaxation times of the nearby tissue, thus overcoming sensitivity limitations of the technique. These can be categorized by their planar outcome, *T*_1_ and/or *T*_2_ effects (longitudinal or transverse effect on relaxation time of water protons, respectively [55]). Similarly, longitudinal and transverse relaxivity (r_1_ and r_2_) are a measure of the goodness of a contrast agent for *T*_1_- and *T*_2_-weighted MR imaging, respectively, and indicate the concentration of contrast agent (mM) that is needed to shorten the relaxation time by one second. 

MNPs are a type of MRI contrast agents with multifunctional properties that are considered interesting probes for their co-localization in specific tissues, such as some tumors. Guldris and co-workers [56,57,58] and Keasberry et al. [59] reported that proper designs of iron oxide MNPs can significantly enhance the diagnostic capability of MRI when compared to other nanostructured Fe-based contrast agents currently available. The most common magnetic labels used in vivo are based on gadolinium (Gd) complexes and iron oxide magnetic nanoparticles (Fe_3_O_4_). The latter has already been successfully used in clinical diagnosis as an MRI contrast agent (e.g., Abdoscan^®^, Resovist^®^, Feridex^®^) [60]. Additionally, and in opposition to Gd complexes, iron-based contrast agents have the potential to be used in *T*_1_- or *T*_2_-weighted imaging with better biocompatibility and safety [54]. Likewise, manganese oxide nanoparticles are of growing interest as an alternative to the Gd chelates as *T*_1_ contrast agents [61,62]. 

To date, several works in the literature have attempted the optimization of MNPs as MRI contrast agents to improve their imaging capabilities for cancer diagnosis. Tse and co-workers reported the synthesis of a prostate specific membrane antigen (PSMA)-targeting iron oxide using a solvent evaporation method, which when directly injected into the prostate induced negative contrast visualization in the MRI [63]. The authors noted the great applicability of the MNPs for the detection and localization of prostate cancer as the result of the great increase in image contrast in in vivo experiments. Similarly, Salimi et al. synthesized iron oxide magnetic nanoparticles coated with a fourth generation polyamidoamine dendrimer (G_4_@IONPs). These G_4_@IONPs MNPs, which were synthesized via a co-precipitation method, significantly shortened the transverse relaxation times (*T*_2_) in in vivo MRI imaging of the mice’s liver after the intravenous administration of the G_4_@IONPs MNPs [64]. Gonzalez-Rodrigues et al. followed a different approach and synthesized multifunctional graphene oxide magnetite (GO-Fe_3_O_4_) loaded with doxorubicin to obtain a formulation with dual magnetic resonance and fluorescence imaging capabilities [65]. The synthesis was here achieved via a coupling reaction between 3-aminopropyltriethoxysilane (APTES)-Fe_3_O_4_ nanoparticles and GO in the presence of the coupling agents N’-ethylcarbodiimide hydrochloride (EDC) and N-hydroxysuccinimide (NHS). These GO-Fe_3_O_4_ MNPs exhibited a high r_2_/r_1_ ratio and great potential to be used as a negative MRI contrast agent in vitro in both cervical and breast cancers cell lines (HeLa and MCF-7, respectively). The authors also reported the use of MRI to study the DOX release from the nanocarrier, together with the translocation of the GO-Fe_3_O_4_ into the cancer cells [65]. In their study, the MRI analysis provided extensive information regarding the drug’s spatial-temporal release and the consequent evaluation of the overall therapeutic efficiency. Another study was conducted by Gallo and co-workers using eco-friendly synthesis of MnO_2__CQDs (carbon quantum dots), which showed OFF–ON responsiveness in the presence of redox stimuli for dual MRI/fluorescence imaging applicability [66].

### 2.3. Magnetic Nanoparticles for Cancer Treatment

Hyperthermia. The use of heat as a treatment for cancer was first tested in 1898 by Frans Westermark, who used hot water in an intracavitary spiral tube to treat advanced cervical cancer [67]. In 1957, Gilchrist et al. administered magnetic nanoparticles for the first time with the intention of generating induction heating capable of selectively killing lymphatic metastases [68]. The authors delivered 5 mg of Fe_3_O_4_ per gram of lymph nodes tissue and then applied an alternating magnetic field (AMF) of 15.9–19.1 kAm^−1^ at 1.2 MHz to obtain a temperature rise of 14 °C. The results of the experiments showed a significant cancer cell death rate without side effects to surrounding tissues [68]. Since then, different methods have been developed to deliver heat as a system for cancer ablation.

This effect, known as hyperthermia or overheating, is a phenomenon where an abnormal higher body temperature occurs (higher than the normal corporal temperature of 37 °C) [69]. This effect can have a variety of origins, including a natural immunological defense mechanism (fever), designed to increase the body’s temperature when suffering an infection [69]. Similarly, overheating can be employed for cancer therapy purposes [70]. Conventional hyperthermia, such as radiofrequency or microwave, is here applied as an adjuvant therapy, ultimately exposing tissues to higher temperatures (up 42 °C) that promote cancer cells apoptosis [71]. As mentioned before, cancer is characterized by an intensification of the cells metabolism rate, amongst other changes, that combined with a disorganized vascular system [1] results in an increased sensitivity to hyperthermia (since the ability to disperse heat is diminished) [68,72]. Additionally, hyperthermia increases the susceptibility of cancer cells to other treatments, including chemotherapy and radiotherapy [72]. However, the main problem of classical hyperthermia is the lack of homogeneity in the heat distribution profile, which can cause harmful side effects in the bordering healthy tissues. Such problems highlight the need to control the temperature increase [73]. 

An alternative that can allow the control of the temperature is the use of tough, magnetic nanoparticles as generators of local heat in specific areas. When an external AMF is applied to generate heat, the approach is called magnetic hyperthermia [74]. Magnetic hyperthermia is a non-invasive treatment where, in the presence of an AMF, magnetic material can transform electromagnetic radiation into thermal energy. Nearby cancer cells heat up to ideally result in tumor ablation [51,75]. Furthermore, intravenous administration of MNPs allows their accumulation on tumorous tissues via passive (by the enhanced permeability and retention (EPR) effect) and potentially active mechanisms (where the MNP surface possesses specific ligands for the surface receptors present in cancer cells) [76]. This accumulation can enable the repetition of posterior AMF treatments with no further MNP administration [33,75]. Additionally, the incorporation of chemotherapeutic drugs inside the formulation allows a synergistic combination of magnetic hyperthermia and chemotherapy, which can overcome some of the concerns related to the magneto-thermal conversion efficiency in vivo (such as degradation of magnetic susceptibility or their inherent absorption under AMF) [77]. 

Rego et al. evaluated the performance of aminosilane-coated superparamagnetic iron oxide nanoparticles as a magnetic hyperthermia treatment in a glioblastoma tumor model. A C6 cell model was evaluated in vitro, whilst Wistar rats were implanted by stereotaxis with C6 cells via stereotaxis for their in vivo evaluation. The authors applied an AMF of 874 kHz and 200 Gauss (20 mT) and observed a 52% and 32.8% in vitro and in vivo cancer cell death, respectively [78]. It is important to highlight that the allowed electromagnetic field that can be applied to living organs should not exceed an upper limit given by the product *H*·*f* = 4.5 × 10^8^ Am^−1^s^−1^ (according to the Brezovich criterion [79]) or *H*·*f* = 5 × 10^9^ Am^−1^s^−1^ (according to Herg et al. [80]).

Similarly, in a recent study, Kandasamy et al. synthesized hydrophilic and surface-functionalized superparamagnetic iron oxide nanoparticles (SPIONs). The synthesized SPIONs were functionalized in situ with short-chained molecules, including 1,4-diaminobenzene (14DAB), 4-aminobenzoic acid (4ABA), and 3,4-diaminobenzoic acid (34DABA). Moreover, their combination with terephthalic acid (TA)/2-aminoterephthalic acid (ATA)/trimesic acid (TMA)/pyromellitic acid (PMA) molecules was explored. The results showed that only the 4DAB-, 4ABA-, 34DABA-, and 4ABA-TA-coated SPIONs presented higher magnetization values than free SPIONS. More specifically, 34DABA-coated SPIONs-based aqueous ferrofluid (AFF, 0.5 mg mL^−1^) showed a faster thermal response and achieved the therapeutic temperature of 42 °C, ultimately having a higher cytotoxic efficiency (61–88%) in HepG2 liver cancer cells [81]. Table 1 summarizes other representative biological studies that have applied MNPs hyperthermia in cancer context.

Chemotherapeutic drug delivery. Chemotherapeutic agents target cells at different phases of cell cycle, which directly or indirectly inhibit the uncontrolled growth of cancer cells [86]. However, the small molecules’ lack of specificity and selectivity towards the cancer tissue can also promote damage to healthy cells, as stated earlier [6,7,8,9,10]. MNPs as a drug delivery system are a potential solution for the delivery of drugs to the desired specific sites. These systems can promote a controlled drug release over time, which provides more efficient therapy for the patient [33] without promoting an overdosage of the drug and associated side effects [87,88]. The drug release from MNPs could present a constant profile (ultimately maintaining a constant concentration for a certain time) or a sigmoidal drug release, reaching a maximum concentration [88]. The use of MNPs as a chemotherapeutic vehicle has been studied [33] since the 1980s, and since then different formulations have been described that incorporate drugs such as DOX [89], paclitaxel (PTX) [90], and methotrexate (MTX) [91] as safer and potential alternatives for the treatment of different cancer types.

In MNPs, these therapeutics can be found either as part of the coating of the nanoparticles (maintained through interactions formed with the surface-active functional groups of the MNPs) or encapsulated/embedded inside them. Both approaches, and especially the latter, can help protect the healthy cells and tissues against the bioactivity of the chemotherapeutic drugs needed to combat cancer. The specific activation of the magnetic nanocarriers under particular conditions after reaching the cancer area can then promote the release of the loaded drugs in the tumor microenvironment. For instance, AMF-generated heat (magnetic hyperthermia) and pH (as the tumor microenvironment has a lower pH than normal physiological values [92]) [93] have been successfully employed as MNP-activation stimuli. Reports of MNPs sensitive to both stimuli have also been reported by Yu et al. [94]. Here, Fe_3_O_4_@SiO_2_ coated with mPEG-poly(l-asparagine) MNPs showed sensitivity to both stimuli (temperature and pH) and as a result displayed an increased DOX release in the tumor region [94]. Similarly, a recent work developed nanocarriers based on an Fe/Mg-carbonate apatite (Fe/Mg-CA) nanoparticles formulation, where different concentrations of Fe^+3^ and Mg^+2^ were used under specific pH to trigger the release of the loaded DOX. The biodistribution study was performed ex vivo; here, both nanoparticles promoted the accumulation of DOX in breast tumors whilst also causing a bigger cytotoxic effect on the cancer and a half-life circulation improvement when compared to the free drug [89].

Applying an AMF as a stimulus for the activation of the drug-loaded MNPs can create a synergistic cytotoxic effect on cancer, where the sum of the parts (the chemotherapy and the magnetic hyperthermia) can cause a bigger phenotypic effect than the individual treatments, as demonstrated by diverse research groups. For example, for the treatment of primary central nervous system lymphoma (PCNSL), Dai et al. [91] used six experimental groups (control, Fe_3_O_4_, MTX, Fe_3_O_4_@MTX, Fe_3_O_4_ with hyperthermia, and Fe_3_O_4_@MTX with hyperthermia) and observed an increase in the apoptosis rate in vitro for the combinatorial treatment when compared to the other groups used. In their in vivo evaluation, the same combination managed to inhibit more the tumor growth when compared to the rest of groups used, as well as managed to decrease the overall tumor cell numbers as measured by H&E staining (hematoxylin and eosin staining). Their results highlight the advantages of this dual treatment in oncology [91]. Other examples are shown in Table 2, which summarizes other similar studies involving chemotherapeutics with or without the application of magnetic hyperthermia or photothermic conditions.

### 2.4. Magnetic Nanoparticles for Theranostic Applications

**Figure 3:**
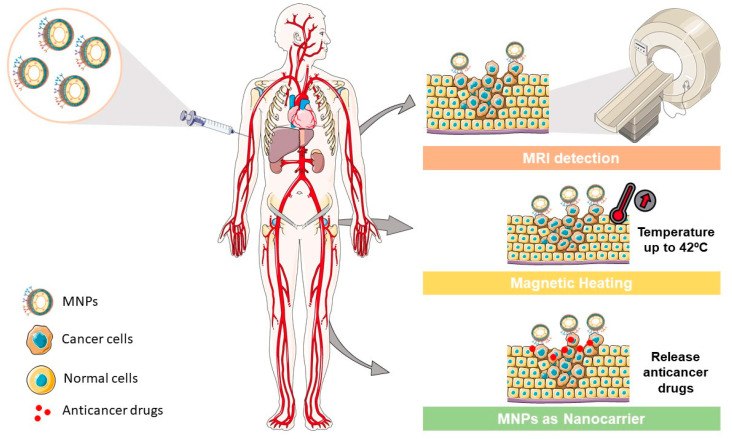
MNP applications in different cancer areas. MNPs could be used as (i) contrast agents to enhance the MRI detection in cancer diagnosis, as (ii) generators for magnetic heating in specific regions such as solid tumors, and as (iii) nanocarriers to deliver specific drugs in cancer treatment.

MNPs have the potential to be used as theranostic platforms in the cancer research field. A theranostic platform combines diagnostic and therapeutic capabilities in the same formulation, enabling efficient tumor targeting, treatment, and therapy response monitoring (or image-guided therapeutics, the visualization of tissue images before, during, and after the treatment) [33]. This combination can help tailor the therapy requirements for each patient within an individualized therapeutic strategy design, with a greater probability of a positive outcome and, at the same time, reduced side effects (Figure 3) [27]. 

Following this path, Abedi et al. [101] synthesized MNP as theranostic platforms by combining modified magnetic mesoporous silica nanoparticles (MMSNs) with imidazoline groups (MMSN-Imi) conjugated with cisplatin (Cis-Pt). The nanoparticles displayed high r_2_/r_1_ reflexivity values and a growth inhibition of ovarian carcinoma cells through apoptosis and necrosis induction, confirming their theranostic applicability in cancer treatment and control [97]. Zhang et al. [98] followed a similar approach by designing an LDH-Fe_3_O_4_-HA (hyaluronic acid) core–shell loaded with encapsulated DOX. The functionalized surface of the Fe_3_O_4_ nanoparticles granted good colloidal stability and cytocompatibility to the nanoformulation, whilst also displaying high r_1_ values and control over its drug release in a pH-dependent manner. The nanoparticles in in vitro phenotypic activity managed to selectively target B16 melanoma cells. The authors also evaluated the nanoparticles’ theranostic efficiency in vivo, using B16 melanoma tumor-bearing C57BL/6 mice through intravenous injection. In vivo, the data showed a reduction of tumor growth in addition to an enhanced MRI contrast in the functionalized nanoparticle-treated group [102]. Table 3 shows other recent studies where theranostic magnetic platforms were designed, synthesized, and evaluated.

To date, several MNPs are in the early stages of clinical trials or in a pre-clinal phase, while different designs have already made it into the clinics for medical imaging and the therapeutic application of solid tumors, such as Feridex IV^®^ (liver and spleen), Lumiren^®^ (bowel), Combidex^®^ (lymph node metastases), and NanoTherm^®^ [36,108].

## 3. Solid Lipid Nanoparticles

**Figure 4:**
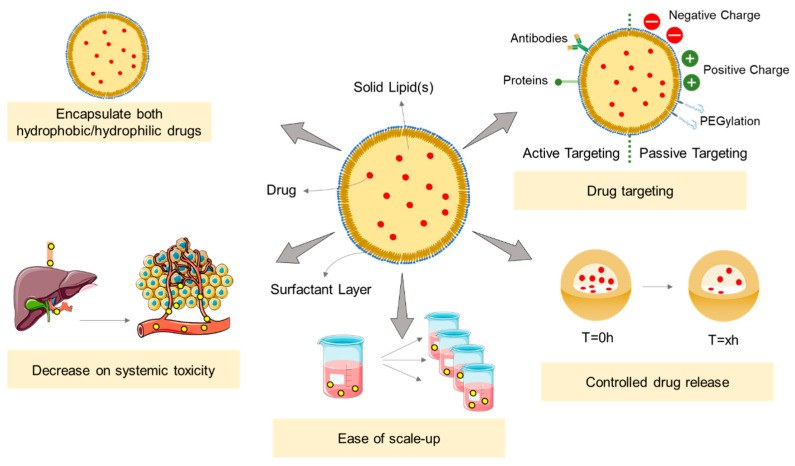
Highlight the applications of SLNs and their major advantages. SLNs could be used as a drug carrier for both hydrophobic and hydrophilic drugs, capable of controlling the drug release, avoiding the “burst effect”, and additionally promoting a target delivery that decreases the systemic toxicity. These nanocarriers could be easily scaled up in a cost-effective manner. Adapted from [116].

SLNs were first remarked upon in the early 1990s [74,109,110,111] as an upgraded alternative of the polymeric, inorganic, and liposomal nanoparticles traditionally used until then as carriers [40]. SLNs are colloidal nanoparticles composed of a lipid matrix, solid at both room and body temperatures [112], and surfactants used as stabilizing and solvating agents (Figure 4) [113]. Different lipid and surfactant compositions can control the size, polydispersity, surface charge, stability, and drug release profile of the formulation [106]. The selection of the lipids can also influence the biodegradability, stability, and affinity by drugs and other elements (metals, dyes, etc.). Commonly, fatty acids such as mono-, di-, and triglycerides, fatty alcohols, and waxes are used for the preparation of SLNs [114]. The small size of the formulations (ranging from 10 to 1000 nm), the large surface-to-volume ratio, and the high drug encapsulation efficiency are the key advantages of SLNs. Additionally, these formulations can potentiate the therapeutic effectiveness of hydrophobic pharmaceuticals [36] by improving their bioavailability, protection from biodegradation and clearance by the reticuloendothelial system (RES), and controlling the drug release rate [115].

### 3.1. SLNs as Drug Delivery Systems

The design of the SLN is the starting point for its development as a potential nanocarrier. For the synthesis of the SLNs, a high-pressure homogenization technique (HPH) methodology has been developed and amply used because of its easiness, efficacy, and relatively low cost [37]. Microemulsions, solvent emulsification method, solvent evaporation or diffusion, and double emulsion techniques have also been used for the preparation of the formulations [37,38,113,117]. However, some of these techniques have drawbacks and limitations, including—for HPH methodology—the mechanic stress applied to the final formulation. Similarly, other techniques depend on a recrystallization step that can reduce the effectiveness of the drug loading (which, however, can be overcome using a heterogenous lipid phase [92]) [118]. 

SLNs formulations are already approved by the FDA and included in the “Generally Recognized As Safe” (GRAS) list. They are recognized as safe to be administered via different routes including intranasal [119], by inhalation [120], intravenous [121], subcutaneous [122], rectal [123], oral [124], ocular [125], and intramuscular [126]. SLNs’ design empowers the biodistribution pharmacokinetics of the intended drugs, improving the drug treatment effectiveness by overcoming the MDR [127]. Additionally, the possibility to modify the SLNs’ surface enhances the capability to overcome biological barriers to target cancer cells with minimal side effects [38] and decrease the initial rapid drug release, called the “burst effect” [37] (major drawback of the drug delivery systems since they could expose the patient to a drug overdose [128]). Identical to what happens with MNPs, coating the SLNs with PEG avoids the rapid immune system cell uptake of these nanocarriers and increases their circulation time [37,38,118,128]. The effect of the functionalization with a PEG-coating was evaluated by Arduino et al., who observed an enhanced ability of the formulation to cross the blood–brain barrier and, consequently, the accumulation of the encapsulated drugs in the brain [129]. Dhiman et al. applied a different approach by synthesizing PEGylated SLNs to enhance the pharmacological profile of the drug in a pathological cardiac hypertrophic model. Their data showed an increase in the circulation time of the PEG-coated nanoparticles and a significant preclusion of the cardiac hypertrophy when compared to the free drug [130].

**Figure 5:**
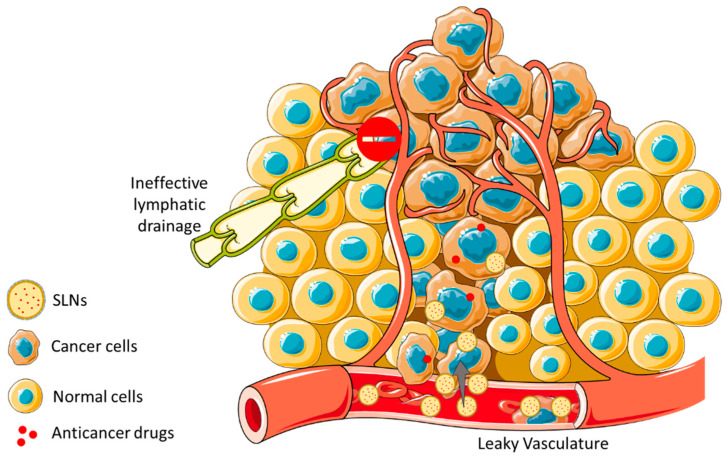
Schematic illustration of the EPR effect and nanoparticles uptake through size across cancerous tissues. EPR effect promotes an increased accumulation of nanoparticles in cancer cells facing normal cells, due to the leaky vasculature within the tumor region being allied to a dysfunctional lymphatic system.

The therapeutic effect of the encapsulated drug is potentially more efficient when the SLNs selectively deliver the drug to its specific site of action. However, the effective accumulation of nanoparticles in solid tumors depends also on the tumors’ microenvironment characteristics as well as the nanoparticles’ physicochemical properties. It has been debated that the EPR effect can hypothetically cause the passive accumulation of nanoparticles, liposomes, or other carriers and macromolecules in tumors because of the enhanced vascular permeability and poor lymphatic drainage surrounding the tumors [131]. This is a consequence of the tumor’s growth requirements, which demands and consumes a high and continuous supply of nutrients and oxygen to be able to sustain its uncontrolled proliferation (Figure 5). To accomplish this, the malignant cells secrete proteins and growth factors, such as fibroblast growth factor (FGF) and vascular endothelial growth factor (VEGF), to induce new blood vessels in a process called angiogenesis, which is one of the hallmarks of cancer [132,133]. The rapid generation of new capillaries in addition to a lack of vasculature supportive tissue (basal membrane) can form an abnormal vessel architecture, with endothelium gaps of diameters between 200 nm to 2 µm of size [134]. Due to this situation, the circulating nanoparticles can easily reach the tumor region through the gaps located in the surrounded blood vessels because of their characteristic small sizes compared to the pore size (<200 nm) [46,131,134,135,136]. In conjugation with an enhanced permeability, an enhanced retention can also be observed due to the deficiency of the lymphatic system. This is because the nanoparticles (characterized by a larger hydrodynamic size) are incapable of returning to the surrounding capillaries, which ultimately increases their retention time in the tumor [136,137,138].

SLNs can also accumulate in the tumor regions through active delivery mechanisms. For this, the SLNs’ surface are functionalized with ligands that can selectively recognize overexpressed receptors on the surface of cancer cells and, ultimately, be translocated inside the cells [136,138]. Consequently, the selective delivery of the pharmacologically active compounds to the tumor can reduce the toxicity and harmful side effects on other healthy cells [46,137,138]. Using these ideas, Rosière and co-workers [139] developed an SLN based on a folate-conjugated copolymer of PEG and chitosan (F-PEG-HTCC) with paclitaxel encapsulated within. In vitro studies with the functionalized SLN showed a decrease of the IC_50_ (half-maximum inhibitory concentration) in overexpressed folate receptor (FR) cell lines in comparison with healthy cell lines with a normal expression of FR. In vivo studies were conducted using female CD1 and BALB/c mice intrapulmonary implanted with M109-HiFR lung cells. Developed nanoparticles were administered to mice through the endotracheal route to perform pharmacokinetic studies. Data demonstrated an enhanced penetrability and prolonged lung residence of the drug-loaded SLNs [139]. Hyaluronic acid is another ligand commonly used as a functionalization moiety for active targeting, as several tumor types are characterized by the overexpression of its receptors (CD44 and CD168). In vitro results obtained by Campos et al. [140] showed enhanced targeting cellular uptake with time/dose-controlled delivery when using a chitosan and hyaluronan (HA)-coated SLN. Their results pointed to an improvement of the chemotherapeutic efficiency [140]. Similarly, SLNs loaded with methotrexate and functionalized with carbohydrates (fucose) were synthesized by Garg and co-workers [141]. In vitro results showed an increase in cytotoxicity against the MCF-7 cancer cell line in comparison to the free drug. Furthermore, in vivo studies were performed using DMBA-induced breast cancer in female Wistar rats. Nanoparticles were intravenously injected into rats and results showed an accumulation of the functionalized SLNs in the tumor microenvironment, which ultimately was associated with an increase in the efficiency of the antitumor treatment. 

### 3.2. Solid Lipid Nanoparticles in Cancer Treatment

**Figure 6:**
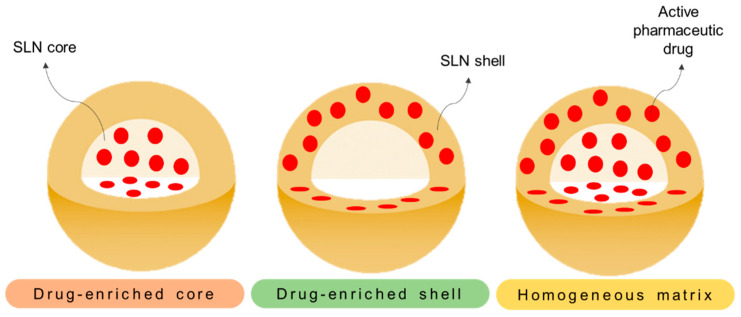
Different representative models of SLNs. On the different models, the drug distribution is represented across (i) the core (drug-enriched core), (ii) the surfactant shell (drug-enriched shell), and (iii) through the core and shell (homogeneous matrix).

As drug nanocarriers, SLNs enable the encapsulation of hydrophobic and hydrophilic drugs (a detailed review on the hydrophilic drug encapsulation can be consulted in [142]) through three potentially distinct manners [76,138]. These can be: (i) dispersed homogeneously in the lipid matrix, (ii) dispersed throughout the shell (surfactant layer), and (iii) incorporated in the core (Figure 6). Several studies have already verified the efficient incorporation of different chemotherapeutic drug types [143,144,145,146] and their evaluation in a wide range of cancers. 

For breast cancer, Xu and colleagues [147] studied the applicability of paclitaxel-loaded SLNs in a drug-resistant breast cancer cell line (MCF-7), whilst Eskiler et al. observed an enhanced anticancer activity of tamoxifen (Tam)-loaded SLNs by inducing apoptosis in both MCF-7 and MCF-7 Tam-resistant cell lines [148]. In the latter, a healthy breast control cell line (MCF-10A) was also used and showed no damage after treatment, validating their use as selective formulations that can even overcome Tam resistance. 

Glioma (brain cancer) has also been targeted with SLNs in some studies to improve the treatment outcome. Marslin et al. used an SLN encapsulated with albendazole (ABZ) [149] and observed an in vitro biphasic release of the drug, where 82% of ABZ was released in 24 h, in addition to an increase of its cytotoxicity and drug uptake in U-87 MG cells compared to the free drug [149]. 

In a similar approach for lung cancer studies, docetaxel (DTX)-loaded SLNs showed, in in vitro studies, a better controlled drug release and an overall activity gain of 100-fold in comparison with the free-drug-treated control in 4T1 cells. Considering the improvement in cellular uptake, SLN-DTX significantly accumulated in cancer cells associated with an induction of cellular apoptosis. Subsequent in vivo studies showed a reduction of tumor growth with the SLNs treatment, without a detectable systemic toxicity in the mice model employed [150]. 

Other examples can be found in Table 4, which summarizes recent SLN preparations and uses them as potential cancer treatments.

SLN formulations represent an advanced nanocarrier system suitable to provide safer and more efficient anticancer treatments, since they are able to overcome many of the limitations of a free-drug administration. However, SLNs with therapeutic properties are still in the initial stages of research and show very limited clinical translation. Large-scale manufacturing processes (able to preserve the stability of drugs), sterilization, and other fabrication technical issues are still challenges that need to be overcome before commercially available SLN products become a reality [157]. For example, an optimization of the SLNs design is still required when using recrystallization synthetic procedures where a drug expulsion from the system can occur, reducing the drug loading capacity [158,159,160], and where the lack of interactions between the drug and the lipid matrix, as well as their chemical nature and state, could also contribute to the poor drug encapsulation [158]. Furthermore, some studies noted a relatively high percentage (70–99.9%) of water content in the dispersion [37,161]. Despite these particular limitations, SLNs constitute simple, scalable, and cost-efficient drug carriers able not only to encapsulate one or several drug candidates and enable multidrug co-delivery approaches but also to provide a functionalization platform towards specific targeting and accumulation in the tumor region, thus offering an enhanced therapeutic index and reduced systemic toxicity. Beyond the encapsulation of anticancer drugs [146,147,148,149,150,152,153,154,155,156], SLNs have already been used to encapsulate siRNA [162,163], DNA [162], platelet aggregation inhibitors [164], and magnetic particles [164,165]. The latter will be further discussed in the next section.

## 4. Magnetic Solid Lipid Nanoparticles

As aforementioned, SLNs present a broad variety of advantages for the treatment of cancer. Several research groups have focused on the development of these new nanoplatforms, trying to exploit and maximize their benefits [164,165,166,167]. However, somewhat surprisingly, the magnetic material incorporation in the SLNs was not explored until quite recently. 

Different metals and metal derivatives such as iron oxide, gold, and gadolinium [74,83,95,96,97,98,99] have been incorporated in the nanoformulations producing novel platforms with great potential in cancer therapy and tissue imaging. In particular, encapsulated iron oxide and gadolinium have been studied abundantly as magnetic delivery systems that can be guided to tumor regions and/or activated for controlled drug release and cell ablation (magnetic hyperthermia) via an external magnetic field or by endogenous stimuli such as pH changes [168,169,170,171]. In particular, iron oxide nanoparticles are considered biocompatible and safe materials and are the gold standard magnetic nanoparticles in medical research, despite the fact that they are able to cause cytotoxicity from the generation of ROS species via the Fenton reaction, which can lead to the damage of DNA, lipids, proteins, and carbohydrates [171,172].

Magnetic solid lipid nanoparticles (mSLNs) represent a new class of functional nanoplatforms that usually consist of inorganic magnetic nanoparticles incorporated in solid lipid nano-matrices and which have great applicability in the medical field [173,174]. For example, Igartua et al. [173] synthesized a colloidal lipid nanoparticle loaded with magnetite using a warm emulsions methodology. The preliminary small size and high entrapment efficiency of the mSLNs managed to fuse the benefits of both types of nanocarriers (SLNs and MNPs) and overcome their independent application issues. mSLNs have shown an enhanced colloidal and chemical stability and caused lower toxicity in vitro compared to the MNPs alone, as described by Müller and colleagues [175], and in vivo using a immunocompetent mice model as described by García-Hevia L. and co-workers [176]. Other groups developed mSLNs constituted with polylactide/glycolide (PLA/GA) and loaded with several different quantities of magnetite to show a controlled drug release via magnetic heating up to 42 °C [177]. 

**Figure 7:**
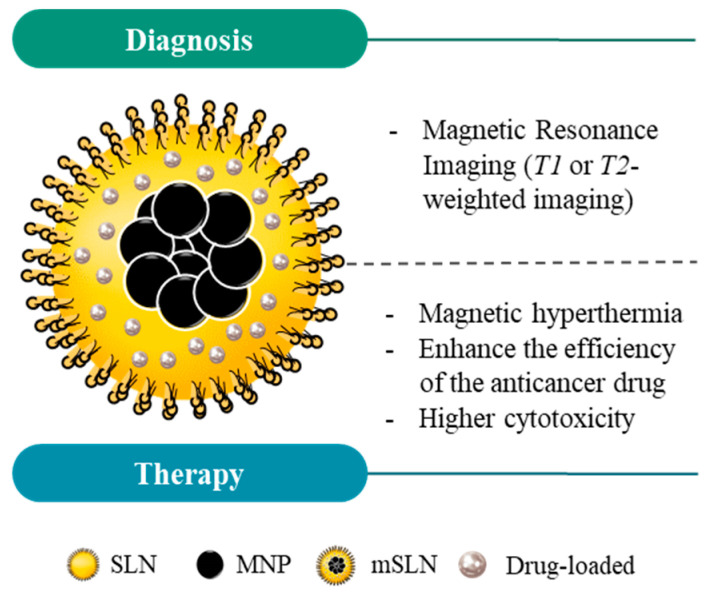
Schematic structure of magnetic solid lipid nanoparticles (mSLNs) and their application in cancer theranostics. Due to the properties of magnetic nanoparticles (MNPs), mSLNs can be used for diagnostic purposes (e.g., MRI application) and cancer therapy via magnetic hyperthermia. Moreover, magnetic hyperthermia in mSLNs offers an extra level of control over the drug release into the region of interest, ultimately increasing the cytotoxicity for cancer cells in comparison with SLNs or MNPs alone.

mSLN synthesis can be achieved through different methodologies, including emulsification ultrasonic dispersion [178], emulsification–diffusion followed by sonication [179], chemical co-precipitation [165,180], and solvent evaporation [181]. The characterization of the resulting mSLNs allows for the elucidation of the structure of the formulation, where the metals can be embedded in the core and/or surface as described by several authors [179,180,181,182]. On the one hand, the metal nanoparticles can be embedded in the lipidic core, where the MNPs’ hydrophobic surface shows chemical affinity by the lipid matrix to yield mSLNs. For the mSLN surface, different surfactants can be used during the synthesis to confer colloidal stability and solvation in water. A schematic representation of mSLNs can be seen in Figure 7.

On the other hand, the metal nanoparticles can be confined in the mSLN surface. Hsu and Su [172] synthesized a new platform that conjugated magnetic heating with a controlled release of the encapsulated drugs (tetracaine) using lipid matrices with γ-Fe_2_O_3_ particles on their surface. γ-Fe_2_O_3_ could then be energized using an external magnetic field, generating enough heat to induce direct thermotherapy as well as to stimulate the release of the loaded drugs in the surrounding tissues. They applied an alternating magnetic field of 60 kA/m at 25 kHz to obtain an increase in temperature of 13 °C in 20 min (up to absolute values of 50 °C). Approximately 35% of the encapsulated tetracaine was released from the mSLNs in 20 min of exposure to the alternating magnetic field [172]. 

Another example of MNPs loaded in SLNs with applicability in controlled drug release was explored by Pang et al. Here, MNPs were first coated with oleic acid and then loaded in the SLNs. Ibuprofen was chosen as a model drug to be also loaded within the mSLNs due to its well-known pharmacological properties. They observed a drug encapsulation efficiency of 80%, and the interaction between the encapsulated MNPs with magnetic hyperthermia application promoted a controlled release from the nanoformulation. They concluded that magnetite-loaded SLNs are viable alternatives as drug delivery systems [178]. Moreover, Oliveira and colleges developed mSLNs with PTX encapsulated via the emulsification–diffusion method. The data showed a 67% encapsulation efficiency, as well as an in vitro drug release rate increase when the temperature was raised from 25 to 43 °C by magnetic hyperthermia. They concluded that the lipid layer played a key role in the controlled drug release mechanism in response to a temperature increase. Similarly, they demonstrated that PTX-loaded mSLNs are promising systems to increase the drug bioavailability, potentially improving future cancer treatments [179]. Using the same approach, Abidi et al. observed a gradual release of albendazole from mSLNs, which reach 84% after 36 h. Their data confirmed these mSLNs as fast and high-efficiency drug delivery systems [183].

Recently, Ahmadifard and co-workers also developed chitosan-coated mSLNs, loaded with letrozole (LTZ), via a modified solvent evaporation–ultrasonic combination method. With this system, 90.1% of the drug was encapsulated, whereas 50% was released after application of a low-frequency pulsed magnetic field (LFPME) at 50 Hz for 1 h, in comparison with the non-LFPME application where the same amount of drug was released in 12 h. Similar to previous reports, their results demonstrated a promising strategy to induce a localized temperature through a magnetic field and a control of chemotherapy treatment in drug-resistant cancers via LTZ release from a nano delivery system [180].

Ghiani et al. synthesized a novel nano-sized contrast agent composed of gadolinium (III) complexes on the surface of solid lipid nanoparticles with a particle size around 50 nm. The developed paramagnetic solid lipid nanoparticles (pSLNs) demonstrated good stability. For MRI studies, IGROV-1 ovarian carcinoma-bearing BALB/c nu/nu mice were used. In vivo MRI revealed an enhancement of the *T*_1_ signal in the tumor region, in particular when folate, used as a targeting ligand, was used to functionalize the nanoparticles’ surface (through intravenous injection). Biodistribution studies in C57BL/6 mice showed an accumulation of pSLNs in the liver, highlighting the need for adjusting the approach in order to enhance the rate of hepatic clearance [184]. 

A recent published work by Rocha et al. describe the synthesis of a novel hybrid magnetic nanocomposite (mHNCs-DOX) which simultaneously incorporates a chemotherapeutic drug (DOX), superparamagnetic iron oxide NPs as a *T*_2_-contrast agent (Fe_3_O_4_) and paramagnetic manganese oxide NPs (MnO) as a *T*_1_-MRI contrast agent [185]. Dual *T*_1_/*T*_2_ MRI performance and additional thermo-chemotherapy capability were observed in vitro in triple-negative breast carcinoma cells (Hs578t cancer cell line) [185]. Table 5 further summarizes representative studies involving mSLNs for cancer treatment/theranostics.

Altogether, the mSLNs have been demonstrated to be promising tools because of their good biocompatibility [171,172,179], improvement of thermo-responsiveness compared to SLNs [168], efficiency in targeting tumors [174,181], and their high drug encapsulation efficiency. Furthermore, these nanosystems allow the application of magnetic hyperthermia as a means to provide thermal therapy and control drug release [164,172,181], in addition to being used as MRI contrast agents [174,181]. Still, there are only few studies involving tests in vivo, highlighting the need to validate the performance of these nanocarriers in more biological complex systems.

## 5. Conclusions

In the last decades, medical nanoformulations have gained value in the biomedical field. Over these years, different materials have been used to form nanoparticle-based carriers including inorganics, organics, hydrogels, micelles, dendrimers, solid lipids, and other materials or combinations of them. Depending on the material, a variety of properties for diverse purposes can be achieved. Cancer theranostics is a ceaselessly growing field and clear target of nanoparticle applications, where numerous nanomaterial-related fabrication and functionalization techniques have been developed with relative success. 

In this review, we analyzed the state-of-the-art MNPs, SLNs, and mSLNs, including their features, advantages, and disadvantages, as well as the most recent works concerning their application in several cancer types. The main objective in this area has been to improve cancer diagnosis and treatment by maximizing the efficiency of contrast agents and therapeutic agents.

## Figures and Tables

**Table 1:** Studies using magnetic nanoparticles (MNPs) for magnetic hyperthermia treatment in cancer.

MNP (Particle Size) + Surface Modification	Treatment + Cancer Model	Results	Ref
SPIONs (250 nm) were coated with targeted CXCR4.	Treatment: 869 kHz and 20 kA·m^−1^ for the first 30 min of the experiment, followed by another 30 min at 554 kHz, and 24 kA·m^−1^. Cancer model: glioblastoma (LN229) and normal kidney cells (HK-2).	In vitro, the targeted treatment conjugated with MH strategy showed a lethal outcome of, approximately, 100% for LN229 cancer cells after 72 h of treatment. The safety profile of NPs was confirmed by the minimal cytotoxicity observed in control group (JK cells—HK-2 cell line).	[82]
IONPs (not specified) were coated with DMSA and conjugated with Gem and the pseudo-peptide NucAnt (N6L).	Treatment: H = 15.4 kA m^−1^; *f* = 435 kHz. Cancer model: pancreatic cancer model (BxPC-3 and PANC-1 cancer cell lines). Athymic nude mice were subcutaneously injected with 2 × 10^6^ BxPC-3 cells.	Combined chemotherapy and treatment with NPs-based MH showed increased cytotoxicity and cell death in vitro (~90% of viable cells compared to approximately 10% when no MH was applied). In vivo, Gem MNPs and the hyperthermia therapy managed to cause an almost complete tumor remission in mice xenografts (at day 28) when compared to the groups receiving only the mono-modal MNP therapy or just the hyperthermia.	[83]
IONPs (46 nm). Fe_3_O_4_@Au MNPs were prepared and loaded with C225.	Treatment: I = 30 A; *f* = 230 kHz. Cancer model: glioblastoma cancer model (U251 cancer cell line). Male and female Balb/c nu/nu nude mice were subcutaneously injected with 2 × 10^6^ U251 cells.	The combined triple therapy decreased, in vitro, cell viability with a high rate of apoptosis via caspase-3, caspase-8, and caspase-9 expression upregulation. In vivo, a significant tumor growth inhibition (approximately 95% of tumor remission) was measured compared to the control groups.	[84]
SPIONs (100 nm) were modified with anti-CD44 antibody.	Treatment: I = 50 A; *f* = 237 kHz. Cancer model: head and neck squamous cell carcinoma stem cells model (Cal-27 cancer cell line). Male Balb/c nude mice were subcutaneously injected with 5 × 10^7^ Cal-27 cells.	CD44-SPIONPs exhibited good biocompatibility and a programmed cell death in cancer stem cells after an AMF application. In vivo, 33.43% of tumor growth inhibition was observed on the treated group.	[85]
^225^Ac SPIONs (10 nm) were attached the attachment of CEPA and transtuzumab to the surface.	Treatment: magnetic flux density from 100 to 300 G and frequency range of 386–633 kHz. Cancer model: ovarian cancer model (SKOV-3 cancer cell line).	^225^Ac@Fe_3_O_4_-CEPA-trastuzumab showed a high cytotoxic effect towards SKOV-3 ovarian cancer cells expressing the HER2 receptor, in vitro.	[17]

IONPs: iron oxide nanoparticles; DMSA: dimercaptosuccinic acid; C225: cetuximab; MNPs: magnetic nanoparticles; Gem: gemcitabine; SPIONs: superparamagnetic iron oxide nanoparticles; AMF: alternating magnetic field; MH: magnetic hyperthermia; ^225^Ac: actinium-225; CEPA: 3-phosphonopropionic acid; NPs: nanoparticles; CXCR4: chemokine cell surface receptor 4.

**Table 2:** Studies using magnetic nanoparticles (MNPs) as drug delivery systems for cancer therapy.

MNP (Particle Size) + Surface Modification	Treatment + Cancer Model	Results	Ref
SPIONs (12 nm). SPIONs were coated with a DMSA, MF66, and covalently functionalized with (i) DOX (MF66-DOX), (ii) pseudopeptide NuCant (MF66-N6L), and (iii) with both (MF66-DOX-N6L).	Treatment: DOX + AMF (H = 15.4 kA/m; *f* = 435 kHz). Cancer model: breast cancer model (BT474 cell line). Female athymic nude mice were subcutaneously injected (on rear backside) with 2.0 × 10^6^ BT474 cells.	The thermo-chemotherapeutic treatment favors the tumor regression in 50% comparatively to control group in vivo (between day 6 and day 17). MF66-DOX-N6L plus hyperthermia application increased their internalization in cancer cells and enhanced in 90% the cytotoxic effect in vitro, comparatively to control group.	[95]
IONPs (112 nm). MnFe_2_O_4_ MNPs were synthesized and were encapsulated in PTX loaded thioether-containing ω-hydroxyacid-co-poly(d,l-lactic acid) (TEHA-co-PDLLA).	Treatment: PTX + AMF (25 mT; *f* = 765 kHz). Cancer model: colorectal cancer model (Caco-2 cell line) + human mesenchymal stem cells derived from adipose tissue.	In vitro experiments showed that NPs were able to sustain PTX release for up 18 days. Moreover, NPs showed great anticancer activity in a dose-dependent manner with low toxicity toward the primary human stem cells derived from adipose tissue.	[96]
IONPs (122 nm). IONPs were modified with a layer of di-carboxylate polyethylene glycol and carboxylate-methoxy polyethylene glycol. Then, IONPs were coated with silica, obtaining PEGylated silica-coated IONs (PS-IONs).	Treatment: DOX + CDDP. Cancer model: breast cancer model (MCF7 cell line); mouse fibroblast cell line (L929).	NPs showed a dual stimuli-triggered release behavior. A release rate of 69% and 84%, for DOX and CDDP, respectively, was measured during the first 30 h in an acidic environment under photothermal conditions. PS-IONs demonstrated potent antitumor activity in vitro, which was significatively enhanced when exposed to low-power near-IR laser irradiation.	[97]
IONPs (non-mentioned). Surface modification is not mentioned.	Treatment: ferumoxytol. Cancer model: mouse mammary tumor virus—polyoma middle T antigen—MMTV-PyMT; MDA-MB-468). Human fibrosarcoma cells (HT1080); murine macrophages (RAW264.7); human dermal fibroblasts (PCS-201-012); human umbilical vein endothelial cells (HUVECs). Female FVB/N were injected with 2.3 × 10^6^ MMTV-PyMT cancer cells.	Ferumoxytil NPs caused tumor growth inhibition by increasing caspase-3 activity. Moreover, macrophages exposed to the NPs enhanced mRNA transcription associated with pro-inflammatory Th1-type responses. In vivo, IONs significantly inhibited the growth of subcutaneous adenocarcinomas compared to controls (tumor size reduction of 53% at day 21), as well as the development of liver metastasis. Additionally, NPs allowed its use as *T*_2_-weighted image for tumor imaging.	[98]
IONPs (20 nm). Surface modification is not mentioned.	Treatment: AT. Cancer model: lung cancer model (A549 and H1975) and human normal lung epithelial cells (BEAS2B); mouse normal liver cells (AML12); rat normal liver cells (BRL3A). Male athymic nude mice were subcutaneously injected with 5 × 10^5^ A549 and H1975 into the dorsal flanks.	AT-MNPs demonstrated inhibition in cancer viability (less than 50% viable cells), whilst displaying no toxicity in vivo. AT-MNP treatment intensified the non-small-cell lung cancer apoptosis, activating the caspase-3 route and downregulating the anti-apoptotic proteins Bcl2 and BclXL, in addition to upregulating the proapoptotic Bax and Bad signals.	[99]
SPIONs (165 nm). Surface modification is not mentioned.	Treatment: MTX + AMF (H023.9 kA/m, *f* = 410 kHz). Cancer model: human bladder cancer cell line (T24). Male SCID (BALB/cJHanHsd-Prkdc) were subcutaneously injected with 2 × 10^6^ T24 cancer cells dorsally between the hindlegs.	The results revealed that the relapse-free destruction of tumors was superior when the combination of chemotherapy and magnetic hyperthermia was used (13 days post-treatment versus 15 days post-treatment under monotherapy). The authors also observed an impairment of proapoptotic signaling, cell survival, and cell cycle pathways.	[100]

SPIONs: superparamagnetic iron oxide nanoparticles; DMSA: dimercaptosuccinic acid; DOX: doxorubicin; AMF: alternating magnetic field; IONPs: iron oxide nanoparticles; MNPs: magnetic nanoparticles; AT: actein; PTX: paclitaxel; CDDP: cisplatin; MTX: methotrexate.

**Table 3:** Studies using magnetic nanoparticles (MNPs) for cancer theranostics.

MNP (Particle Size) + Composition	Treatment + Cancer Model	Results	Ref
MnO_2_ NPs (107 nm) loaded with poly(N-vinylcaprolactam) nanogels (PVCL NGs) (DOX/MnO_2_@PVCL NG).	Treatment: DOX Cancer model: melanoma cancer model (B16 cancer cell line). In vivo: mouse model of subcutaneous B16 melanoma.	NPs showed interesting biocompatibility properties in addition to redox responsiveness in tumoral tissues. In an in vivo tumor model (with relatively high concentration of GSH), a release of Mn^+2^ from DOX/MnO_2_@PVCL NG occurred that enhanced *T*_1_-weighted MRI. In parallel, the DOX release from the NPs inhibited the tumor growth (1 versus 14 relative tumor growth for dual-treatment and control, respectively).	[103]
Fe_3_O_4_ IONPs (200–300 nm) were synthesized and functionalized with PDA, PEG, and cRGD (Fe_3_O_4_@PDA-PEG-cRGD).	Treatment: DOX + photothermal effect (1 W/cm^2^). Cancer model: colon cancer model (HCT-116 cancer cell line). Male nude mice were subcutaneously injected with HCT-116 cells (5 × 10^6^/mL).	In vitro and in vivo, NPs were capable of targeting tumor cells and promoting the drug internalization. The cytotoxic effect was also significant (survival rate of 25.6% comparatively to control group) whilst the nanocarriers displayed good thermal stability and photothermal conversion efficiency, pH responsiveness, and an enhancement of *T*_2_-MRI contrast. In vivo, the authors observed a decrease in tumor growth around 67% when compared the dual-treatment with the control.	[104]
IONPs (26 nm) were coated with casein (CION) and functionalized with the tumor-targeting ATF of urokinase plasminogen activator and the antitumor drug CDDP (ATF-CNIO-CDDP).	Treatment: CDDP. Cancer model: pancreatic cancer model (MIA PaCa-2 cancer cell line). Female nu/nu mice were injected with 1 × 10^6^ MIA PaCa-2 cells (orthotopic pancreatic tumor model).	NPs promote a *T*_2_-MRI contrast, combined with an improvement of therapeutic effectiveness (0.75 g versus 1.5 g of tumor weight for treated group and control, respectively) and a decrease on harmful side effects in comparison to the free drug.	[105]
SPIONs (260 nm) were coated with FA and ACPP (F/A-PLGA@DOX/SPIO).	Treatment: DOX. Cancer model: human non-small cell lung cancer model (A549 cell line). Normal liver cell (L02 cell line). Male BALB/c nude mice were subcutaneously injected with 3 × 10^7^ A549 cells into the right-rear leg.	F/A-PLGA@DOX/SPIO induced apoptosis in the cancer cells, accelerating the overproduction of ROS. MRI was used to track the NPs in cancer cells (*T*_2_-weighted MRI). In vivo, a reduction in tumor growth was observed (around 67% comparatively to control group), NPs showed a good biocompatibility and long plasma stability, with a capability to induce tumor necrosis, whilst no significant damage or inflammation was detected in healthy organs.	[106]
SPIONs (6 nm) were coated with dextran (FeDC-E NPs).	Treatment: erlotinib. Cancer model: lung cancer model (CL1-5-F4 cancer cell line). Male BALB/c nude mice were subcutaneously injected with 2.5 × 10^6^ of CL1-5-F4 cells.	Theranostic NPs showed a significant therapeutic effect with targeting properties against invasive and migrative cancer cells. These NPs enabled their localization using *T*_2_-weighted MRI. EGFR–ERK–NF-κB signaling pathways were suppressed when after tumors treatment.	[107]

SPIONs: superparamagnetic iron oxide nanoparticles; IONPs: iron oxide nanoparticles; MNPs: magnetic nanoparticles; NPs: nanoparticles; DOX: doxorubicin; PDA: polydopamine; PEG: poly(ethylene glycol); cRGD: cyclic arginine-glycine-aspartate motif; ATF: amino-terminal fragment; GSH: glutathione; MRI: magnetic resonance imaging; CDDP: cisplatin; FA: folic acid; ACPP: activable cell-penetrating peptide; ROS: reactive oxygen species.

**Table 4:** Solid lipid nanoparticles (SLNs) as drug delivery systems for cancer therapy.

SLN (Particle Size) + Surface Modification/Loading	Drug + Cancer Model	Results	Ref
SLNs (200 nm). Surface modification is not mentioned.	Drug: DOX. Cancer model: murine malignant melanoma (B16F10 cells). C57BL/6 mice (12–16 weeks old) were intravenously injected with 1 × 10^5^ B16F10 cells.	In vivo, mice treated with SLNs-DOX, obtained, approximately, a 60% reduction of tumor area when compared to mice treated with free DOX. No significant differences were found in the survival rates or body weight between different treatment groups, indicating no detectable SLPs-DOX in vivo toxicity during the timeframe of these tests.	[151]
PTX-SLN (<200 nm). Surface modification is not mentioned.	Drug: PTX. Cancer model: breast cancer model (MCF-7 cancer cell line).	Xu et al. observed an enhanced anticancer activity of PTX-SLNs, which significantly increased the intracellular uptake (almost 10 ng more of PTX *per* mg of protein comparatively to control) of the drug when compared to the free drug. The results demonstrated that the use of SLNs could efficiently avoid the multidrug resistance mechanisms observed in breast cancer cells.	[147]
SLN-TMZ (279 nm). Surface modification is not mentioned.	Drug: TMZ. Cancer model: melanoma cancer model (JR8 and A2058 cell lines; B16-F10 mouse melanoma cell line). Female C57BL6/J mice were subcutaneously injected with 1 × 10^6^ B16-F10 cells.	NPs showed in vitro and in vivo their ability to target tumor cells and promote drug internalization, reducing the therapeutic dosage needed to be administered in the in vivo model. Here, SLN-TMZ also displayed a higher mice survival rate compared to that obtained using the free drug (increasing from 50 to 100%). Moreover, the in vitro tumor angiogenesis was found to be inhibited (HUVEC method).	[152]
Chol-CUR-SLN (170 nm). Surface modification is not mentioned.	Drug: CUR. Cancer model: breast cancer model (MDA-MB-231 cell line).	In vitro results showed that Chol-CUR-SLN efficiently targeted and accumulated in cancer cells. It also exhibited a higher inhibitory effect on cell viability (20% of higher cytotoxicity in comparison to free drug) and proliferation when compared to free CUR. Chol-CUR-SLN significantly improved the induction of apoptosis (63.87% versus 55.4%) in MDA-MB-231 cells, compared to free CUR.	[153]
SLN-MTX (300 nm) loaded with an ApoE mimicking chimera peptide to actively target the brain.	Drug: MTX. Cancer model: glioblastoma cancer model (F98/Fischer glioblastoma human primary culture).	A reduction of tumor growth (relative tumor growth of approximately 4 versus 10 for treated and control groups, respectively) was observed with SLN-MTX. Moreover, an increase of apoptosis was noted, demonstrating that the developed SLN could be an alternative to conventional therapy.	[154]
TAT PTX/TOS-CDDP SLNs (100 nm) modified with DSPE-PEG and TAT for co-delivery of PTX and TOS-CDDP.	Drug: PTX + TOS-CDDP. Cancer model: cervical cancer model (HeLa cancer cell line). BALB/c nude mice were subcutaneously injected with 1 × 10^6^ of HeLa cells.	TAT PTX/TOS-CDDP SLNs had a slower drug release in comparison with PTX/TOS-CDDP SLNs. Here, the drug release was greatly affected by a lower pH. The in vitro cellular uptake study also showed that tumor cells could uptake more efficiently the TAT PTX/TOS-CDDP SLNs when compared with other SLNs. Moreover, these nanoparticles showed a synergistic effect in the suppression of tumor growth in vivo (inhibition rate of 72.2%) with lower toxicity (calculated by the bodyweight loss during the experiment). Moreover, the formulation increased the drug accumulation in tumor tissue in comparison to the administration of the free drug.	[155]
c-SLN (200 nm). Surface modification is not mentioned.	Drug: FA+ ASP. Cancer model: pancreatic cancer model (PaCa-2 and Panc-1 cell lines). Male SCID mice were subcutaneously injected with 1 × 10^6^ PaCa-2 cells.	In vitro studies demonstrated that NPs with the conjugated treatment effectively inhibited cell growth, inducing apoptosis. The use of the dual treatment loaded in the SLNs presented significantly better results in cell viability assays when compared to the cells treated with the free drugs. The in vivo studies presented a tumor growth suppression of 45% compared to the control group. However, this result was not statistically significant. By performing the immunohistochemistry analysis, an increased expression of pro-apoptotic proteins was detected.	[156]

SLNs: solid lipid nanoparticles; NPs: nanoparticles; PTX: paclitaxel; TMZ: temozolomide; CUR: curcumin; Chol: cholesterol; ApoE: very low-density lipoprotein receptor binding; MTX: methotrexate; DSPE: 1,2-distearoyl-sn-glycero-3-phosphorylethanolamine; PEG: poly(ethylene glycol); TAT: trans-activating transcriptional activator; TOS-CDDP: α-tocopherol succinate-cisplatin prodrug; c-SLN: chitosan-coated solid lipid nanoparticle; FA: ferulic acid; ASP: aspirin; DOX: doxorubicin; HUVEC: human umbilical vein endothelial cells.

**Table 5:** Magnetic solid lipid nanoparticles (mSLNs) as drug delivery systems and theranostic agents against cancer.

mSLN (Particle Size) + Surface Modification	Drug + Cancer Model	Results	Ref
Wax-mSLNs (200 nm). Surface modification is not mentioned.	Drug: DOX. Cancer model: murine melanoma B16f10, Hs578t, and Dox-resistance cell lines (t84 and HCT-15).	Efficacy studies showed that DOX delivery in combination with 1 h of MH promoted a significant cytotoxic effect in vitro in melanoma cell lines compared to a treatment in which no MH was supplied (~5% vs. ~50%, respectively, when using 1 µg DOX/mL of DOX-mSLNs). Similar results were obtained in 3D in vitro using melanoma spheroids. The same dual treatment approach was applied to DOX-resistant cell lines obtaining approximately 40% of cell viability reduction.	[186]
Wax-mSLNs (250–300 nm). Surface modification is not mentioned.	Drug: OncoA. Cancer model: human lung carcinoma cell line (A549 cell line).	mSLNs showed an outstanding performance as a *T*_2_-contrast agent in MRI (*r*_2_ > 800 mm^−1^ s^−1^). In vitro, the combination of co-loaded MNPs and OncoA with MH greatly decreased the cell viability (virtually 0% vs. 53% when performed without MH application) at the same 40 µg OncoA/mL and 25 µg Fe/mL doses).	[187]
Wax-mSLNs (200 nm). Surface modification is not mentioned.	Drug: DOX. MH: 224 kHz, 13 A, 27.6 W for 1 h for in vitro 174.5 kHz, 23 mT for 1 h for in vivo. Cancer model: murine malignant melanoma cells (B16F10 cell line); C57BL/6 mice (8–10 weeks old) were subcutaneously injected in interscapular region of mice with 5 × 10^5^ B16F10 cells.	mSLNs-DOX showed higher cytotoxicity activity than free DOX in the whole range of DOX concentration tested both in vitro and in vivo. In vitro, a remarkable enhanced cytotoxicity was obtained when cells were exposed to the combination of chemotherapy (0.5 µ/mL) and 1 h MH (40% of viable cells vs. 85% without MH). Under a higher incubation concentration of mLNVs-DOX (1 μg DOX/mL), the results showed a cytotoxicity virtually to 100% under a combination of mLNVs-DOX with MH. In vivo, the dual treatment promoted the slowest tumor growth and smallest tumor volume, which was on average 3 and 2.1-fold smaller than the saline and free-DOX groups. Regarding imaging capability, *T_2_-MRI* relaxation times of animal tumors treated with mSLNs were on average over 15% shorter than those of control animals injected only with saline.	[176]
Sor-mag-SLN (250 nm). Surface modification is not mentioned.	Drug: Sor. Cancer model: liver cancer model (HepG2 cell line).	The nanocarriers showed a loading efficiency of 90% and stability in an aqueous environment. Moreover, the developed nanoparticles presented a good cytocompatibility with a high antiproliferative effect against the cancer cells (40% higher in comparison to control group). This effect was associated with the capability of these nanocarriers to be specifically accumulated in the tumor region and the application of a local AMF.	[188]
Mag-SLN (150 nm). Surface modification is not mentioned.	Cancer model: myeloid leukemia cancer model (HL-60/wt cell lines; L-60/adr with MRP1 = ABCC1 over-expression; HL-60/vinc with P-glycoprotein = ABCB1 over-expression), leukemia cancer model (Jurkat T-cells), and glioblastoma cancer model (U251 cell line).	The developed nanoparticles showed promising results in the context of cancer therapy, in particular against drug-resistant cell lines. The mag-SLN revealed higher cytotoxicity against resistance cell lines in comparison to DOX alone when under an AMF. Moreover, the data showed that the cells treated with a dual treatment presented an increase of nuclei fragmentation and condensed chromatin. The mag-SLNs plus MH presented apoptotic and necrotic activities. The authors proposed that the production of ROS was the cause of the higher cytotoxicity observed in the cells treated with the particles.	[168]
LMNV (100 nm). Surface modification is not mentioned.	Drug: TMZ. Cancer model: glioblastoma cancer model (U-87 cell line) and brain-endothelial cell model (bEnd.3 cell lines, an immortalized mouse BEC line).	In vitro results showed that lipid-based magnetic nanovectors presented a good loading capacity with a sustained release profile of the encapsulated chemotherapeutic drug. Moreover, a complete drug release was observed after the exposure to (i) low pH (4.5), (ii) increased concentration of hydrogen peroxide (50 µM), and (iii) increased temperature achieved through the application of an AMF. The authors noted that these nanovectors could be used as a potential hyperthermia agent, since they managed to increase apoptotic levels and decrease proliferative rates when a magnetic field of 20 mT and 750 kHz was applied, increasing the temperature to 43 °C. During in vitro tests, the capacity of LMNVs to cross the BBB was observed, where after 24 h of exposure, 40% of LMNVs were able to translocate inside the glioblastoma cells.	[189]
Gd(III)-loaded pSLNs were modified with with cellular receptors, DSPE-PEG2000-folate.	Cancer model: murine macrophage model (Raw 264.7 cell line), lymphoma cancer model (U937 cell line), and human ovarian adenocarcinoma (IGROV-1 cell line). Female Balb/C nu/nu were subcutaneously injected with 1 × 10^7^ of IGROV-1 cells.	The data showed that pSLNs could effectively internalize in in vitro and in vivo models. Moreover, the authors detected the nanoparticles’ *T*_1_-MRI signal, at least after 30 min post-injection. The cytotoxic studies showed a decrease in cell viability when the loaded Gd(III) concentration increased within the pSLN (below 50% of viable cells). The results also demonstrated that Gd(III)-loaded pSLNs could efficiently target the cancer cells and due to the EPR effect in conjunction with its targeting properties allowed a higher internalization capacity. Moreover, they could be used as a molecular imaging tool. A macrophage uptake experiment in vivo showed that the nanoparticles could avoid the macrophage internalization and circulate for at least 6 h, increasing altogether the tumor uptake. However, the authors noted an excessive accumulation in the liver with slow elimination rates after performing the biodistribution study.	[184]
Sor-Mag-SLNs (300 nm). Surface modification is not mentioned.	Drug: Sor. Cancer model: liver cancer model (HepG2 cell line).	The results showed an increase of the cytotoxic effects of sorafenib. Using an external magnetic field, it was possible to guide and improve the drug effect in the desired area. Quantitative evaluation of cell mortality indicated 95% of cell death compared to the control (5%). Moreover, the authors mentioned that the nanocarriers could be an effective approach to reduce the undesired side effects of chemotherapeutic drugs and improve their pharmacokinetic properties.	[190]
Nut-Mag-SLNs (180 nm) were loaded with fluorescenin-PEG-DSPE (FITC-PEG-DSPE).	Drug: Nut. Cancer model: glioblastoma cancer model (U-87 cancer cell line) and brain endothelial cell model (bEnd.3 cell lines, an immortalized mouse BEC line).	Nut-Mag-SLNs presented a good colloidal stability and could efficiently cross an in vitro blood–brain barrier model. The authors observed that the nanovectors were magnetically activated, enabling their pass through the BBB, and could also deliver the drug loads to glioblastoma cells. Moreover, they observed an enhanced antitumor activity as they obtained a 50% reduction in the metabolic activity with lower drug concentrations. Increased pro-apoptotic activity was also noted. These nanocarriers presented several advantages compared to the free drug in overcoming several limitations in glioblastoma treatments, for instance, (i) Nut-Mag-SLNs could cross the BBB, (ii) Nut-Mag-SLNs had the ability to be magnetically guided to the tumor region, and (iii) the nanoparticles showed a powerful inhibition of cancer cell proliferation while increasing the pro-apoptotic activity.	[181]
mSLNs (180 nm). Surface modification is not mentioned.	Cancer model: colon cancer model (HT-29 cell line).	By applying magnetic hyperthermia, results showed that mSLNs could constantly maintain the maximum temperature achieved (46 °C, in 40 min) during 1 h of exposure to a magnetic field (250 kHz and 4 kA/m). These results translated into a decrease in cell viability after magnetic treatment (up to 52% comparatively to 100% of control group). Interestingly, no cytotoxic effect was observed if only one (but not both) of the components was used alone for treatment.	[165]

Mag-SLN (mSLN): magnetic solid lipid nanoparticles; Sor: sorafenib; MRP1: multidrug resistance-associated protein 1; TMZ: temozolomide; BBB: blood–brain barrier; pSLNs: paramagnetic solid lipid nanoparticles; AMF: alternating magnetic field; DSPE: 1,2-distearoyl-sn-glycero-3-phosphorylethanolamine; PEG: poly(ethylene glycol); EPR effect: enhanced permeability and retention effect; Nut: Nutlin; DOX: doxorubicin; OncoA: oncocalyxone A.
